# ﻿*Liparismacrosepala* (Orchidaceae), a new species from southwest China with its phylogenetic position

**DOI:** 10.3897/phytokeys.210.87033

**Published:** 2022-10-04

**Authors:** Zhengwei Wang, Yi Zhang, Ze Zhang, Xiaochen Li, Zhijin Wu, Lan Yan, Aixian Lu, Chengzhi Xie, Chao Hu, Weichang Huang

**Affiliations:** 1 Eastern China Conservation Centre for Wild Endangered Plant Resources, Shanghai Chenshan Botanical Garden, Shanghai 201602, China Eastern China Conservation Centre for Wild Endangered Plant Resources, Shanghai Chenshan Botanical Garden Shanghai China; 2 Yunnan Yelantang Biological Technology Co., Ltd., Kunming 650114, China Yunnan Yelantang Biological Technology Co., Ltd. Kunming China; 3 College of Art and Landscape Architecture, Fujian Agriculture and Forestry University, Fuzhou 350002, China Fujian Agriculture and Forestry University Fuzhou China; 4 Key Laboratory of Genetics and Germplasm Innovation of Tropical Special Forest Trees and Ornamental Plants, Ministry of Education, College of Forestry, Hainan University, Haikou 570228, China Hainan University Haikou China

**Keywords:** *Liparis* section *Cestichis*, molecular phylogeny, morphology, *matK*, nrITS

## Abstract

A new orchid species, *Liparismacrosepala*, is illustrated and described from Yunnan Province, China, based on morphological and molecular analyses. This plant is characterised by the ovoid-fusiform, slightly compressed pseudobulbs with 4 or 5 leaves with slightly crisped margins on their apical half, dorsal sepal heart-shaped, lip with a bituberculate basal callus and a thickened folded lateral lobe on each side, centrally with one cavity with slightly raised margins, the column with a single pair of broadly triangular, obtuse wings. Maximum Likelihood and Bayesian Inference analyses of combined nrITS and plastid *matK* DNA sequences place this species in section Cestichis.

## ﻿Introduction

The genus *Liparis* Rich. (Epidendroideae, Malaxideae, Malaxidinae) comprises about 320 species distributed worldwide with more than 70 species in China (Pridgeon et al. 1999; Chen et al. 2009; Tian et al. 2015; Huang et al. 2018; Ya et al. 2021). Species from this genus are terrestrial, lithophytic, epiphytic and rarely mycoheterotrophic, with inflorescences laxly or densely many-flowered, lip often reflexed and usually with a basal callus, lacking a spur, column winged at apex and sometimes at base and four pollinia in two pairs (Chen et al. 2009).

During our field surveys in Xishuangbanna, Yunnan, China, an unknown species was found. In this paper, we analysed the morphological differences of the newly-found species and its allied species and the phylogenetic position of the new entity is also discussed, based on molecular evidence from nrITS and plastid *matK*. After careful morphological comparison and phylogenetic analyses, we concluded that this species is new to science.

## ﻿Material and method

### ﻿Morphological observations

Materials of the new species were collected from Xishuangbanna, Yunnan, China during a field expedition. Morphological characters were observed, measured and photographed based on five living individuals under a stereomicroscope (SZX16-6151, Olympus, Japan) and photographed with a digital camera (D750, Nikon, Japan). A voucher specimen, designated as the holotype, was deposited at Shanghai Chenshan Herbarium (CSH). Conservation assessment has been conducted following IUCN guidelines (IUCN 2019).

### ﻿Taxonomic sampling

DNA sequences of nrDNA ITS and plastid *matK* of the new species were sequenced and sequences of the same markers for 82 related species were downloaded from GenBank, including five outgroup species from other subtribes (Table 1).

**Table 1.** Taxon sampling in this study.

	Species Name	nrITS	*matK*
**1**	*Acanthophippiummantinianum* L.Linden & Cogn.	AF521081	AF263618
**2**	*Collabiumsimplex* Rchb.f.	EF670387	AY557200
**3**	*Crepidiumacuminatum* (D.Don) Szlach.	KJ459274	KJ459304
**4**	*Crepidiumbahanense* (Hand.-Mazz.) S.C.Chen & J.J.Wood	MH116611	MH117500
**5**	*Crepidiumbancanoides* (Ames) Szlach.	AB290885	AB290893
**6**	*Crepidiumbrevidentatum* (Schweinf.) M.A.Clem. & D.L.Jones	AB290886	AB290894
**7**	*Crepidiumresupinatum* (G.Forst.) Szlach.	JN114483	JN004403
**8**	*Dendrobiumdixanthum* Rchb.f.	KY966535	KY966825
**9**	*Dieniacylindrostachya* Lindl.	JN114491	JN004422
**10**	*Eriaferruginea* Lindl.	AF521071	AF263660
**11**	*Eulophiagraminea* Lindl.	MH768269	MH767976
**12**	*Liparismacrosepala* Z.W. Wang, Y. Zhang & W.C. Huang	ON642332	ON642331
**13**	*Liparisanopheles* J.J.Wood	AY907075	AY907139
**14**	*Liparisassamica* King & Pantl.	KJ459276	KJ459306
**15**	*Liparisaureolabella* J.D. Ya & Z.D. Han	MN065679	MN065733
**16**	*Liparisauriculata* Blume ex Miq.	AB289458	KF262076
**17**	*Liparisbalansae* Gagnep.-1	KF589874	KF589880
**18**	*Liparisbalansae* Gagnep.-2	KJ459277	KJ459307
**19**	*Liparisbingzhongluoensis* X.H. Jin	MW169041	MW169042
**20**	*Liparisbistriata* E.C.Parish & Rchb.f.	KJ459279	KJ459309
**21**	*Liparisbootanensis* Griff	KJ459280	KJ459310
**22**	*Liparisbracteata* T.E.Hunt	AY907076	AY907140
**23**	*Liparisbrunnescens* Schltr.	AY907098	AY907165
**24**	*Lipariscondylobulbon* Rchb.f.	AY907080	AY907144
**25**	*Lipariscordifolia* Hook.f.	KJ459282	KJ459312
**26**	*Liparisdelicatula* Hook.f.	KJ459283	KJ459313
**27**	*Liparisdistans* C.B.Clarke	KJ459284	KJ459314
**28**	*Liparisdisticha* (Thouars) Lindl.	AY907081	AY907145
**29**	*Lipariselliptica* Wight	KJ459285	KJ459315
**30**	*Liparisfissilabris* Tang & F.T.Wang	KJ459286	KJ459316
**31**	*Liparisfissipetala* Finet	KJ459287	KJ459317
**32**	*Liparisformosana* Rchb.f.	AY907082	AY907147
**33**	*Liparisfujisanensis* F.Maek. ex Konta & S.Matsumoto	EU024936	EU024937
**34**	*Liparisgibbosa* Finet-1	AY907083	AY907148
**35**	*Liparisgibbosa* Finet-2	AY907084	AY907149
**36**	*Liparisglossula* Rchb.f.	KJ459289	KJ459319
**37**	*Liparisguangxiensis* C.L.Feng & X.H.Jin	KF589875	KF589881
**38**	*Liparisjaponica* (Miq.) Maxim.	AY907086	AY907151
**39**	*Lipariskoreana* (Nakai) Nakai	EU017422	EU017444
**40**	*Lipariskumokiri* F.Maek.	AY907087	AY907152
**41**	*Liparislatifolia* Lindl.	AY907088	AY907153
**42**	*Liparislatilabris* Rolfe	KJ459291	KJ459321
**43**	*Liparisliliifolia* (L.) Rich. ex Lindl.	AY907090	AY907156
**44**	*Liparisloeselii* (L.) Rich.	AY907091	AY907157
**45**	*Liparismakinoana* Schltr.	EU017405	EU017428
**46**	*Liparismannii* Rchb.f.	KJ459293	KJ459323
**47**	*Liparismeihuashanensis* S.M.Fan	MF959772	MF959773
**48**	*Liparismengziensis* J.D. Ya & Lei Cai	MN065734	MN065678
**49**	*Liparisnanlingensis* H.Z.Tian & F.W.Xing	AB701346	/
**50**	*Liparisnapoensis* L.Li, H.F.Yan & S.J. Li-1	MT012899	MT019986
**51**	*Liparisnapoensis*L.Li, H.F.Yan & S.J. Li -2	MT012900	MT019987
**52**	*Liparisnervosa* (Thunb.) Lindl.	AY907092	AY907158
**53**	*Liparisnugentiae* F.M.Bailey	AY907093	AY907159
**54**	*Liparisodorata* (Willd.) Lindl.	KJ021033	KJ021029
**55**	*Liparispandurata* Ames	AY907094	AY907160
**56**	*Liparispauliana* Hand.-Mazz.	AY907096	AY907163
**57**	*Liparispetiolata* (D.Don) P.F.Hunt & Summerh.	MW186826	MW187482
**58**	*Liparisresupinata* Ridl.	KJ459297	KJ459327
**59**	*Liparissomae* Hayata-1	MT012898	MT019985
**60**	*Liparissomae* Hayata-2	MT012897	MT019984
**61**	*Liparissootenzanensis* Fukuy.	KJ021034	KJ021030
**62**	*Liparisstricklandiana* Rchb.f.-1	MT012903	MT019990
**63**	*Liparisstricklandiana* Rchb.f. -2	KJ459298	KJ459328
**64**	*Liparissula* N.Hallé	AY907104	AY907171
**65**	*Liparisterrestris* J.B.Comber	AY907105	AY907172
**66**	*Liparistruncicola* Schltr.	AY907106	AY907173
**67**	*Liparisviridiflora* (Blume) Lindl.-1	MT012902	MT019989
**68**	*Liparisviridiflora* (Blume) Lindl.-2	MT012901	MT019988
**69**	*Malaxisbrachypoda* (A.Gray) Fernald	AY907108	AY907175
**70**	*Malaxismonophyllos* (L.) Sw.	MW181626	MW187483
**71**	*Malaxissoulei* L.O.Williams	AY907119	AY907186
**72**	*Malaxisabieticola* Salazar & Soto Arenas	AY907129	AY907196
**73**	*Oberoniaacaulis* Griff.	KY242066	KY241943
**74**	*Oberoniabrunoniana* Wight	JN114623	JN004516
**75**	*Oberoniaequitans* (G.Forst.) Mutel	AY907130	AY907198
**76**	*Oberoniaheliophile* Rchb.f.	AY907131	AY907199
**77**	*Oberoniairidifolia* Roxb. ex Lindl.	AY907132	AY907200
**78**	*Oberoniamucronata* (D.Don) Ormerod & Seidenf	JN114640	JN004534
**79**	*Oberonianeocaledonica* Schltr.-1	AY907133	AY907201
**80**	*Oberonianeocaledonica* Schltr.-2	AY907134	AY907202
**81**	*Oberoniapadangensis* Schltr.	AY907135	AY907203
**82**	*Oberoniawappeana* J.J.Sm.	AY907138	AY907206
**83**	*Oberonioidespusillus* (Rolfe) Marg. & Szlach.	KJ527610	KJ459302

### ﻿Phylogenetic analyses

DNA sequences were aligned using the MAFFT programme in Geneious v. 2020.2.4 (https://www.geneious.com, accessed on 10 March 2021). Phylogenetic analyses were conducted using Maximum Likelihood (ML) and Bayesian Inference (BI) in RAxML v.7.0.4 (Stamatakis 2006) and MrBayes v.3.2.6 (Huelsenbeck and Ronquist 2001; Ronquist et al. 2012), respectively. The appropriate DNA substitution model under AIC criteria was estimated using jModelTest 2.1.10 (Posada 2008). ML analyses were conducted with bootstrap values calculated by running 1,000 replicates. For BI analysis, four chains were run with random initial trees, each for 1,000,000 generations, until the average standard deviation of the split frequency values was less than 0.01 to ensure convergence, sampling trees every 1,000 generations. After the first 20% of samples were discarded as burn-in, the remaining replicates were used to estimate the posterior probabilities.

## ﻿Results

### ﻿Phylogenetic analyses

**Figure 1.**
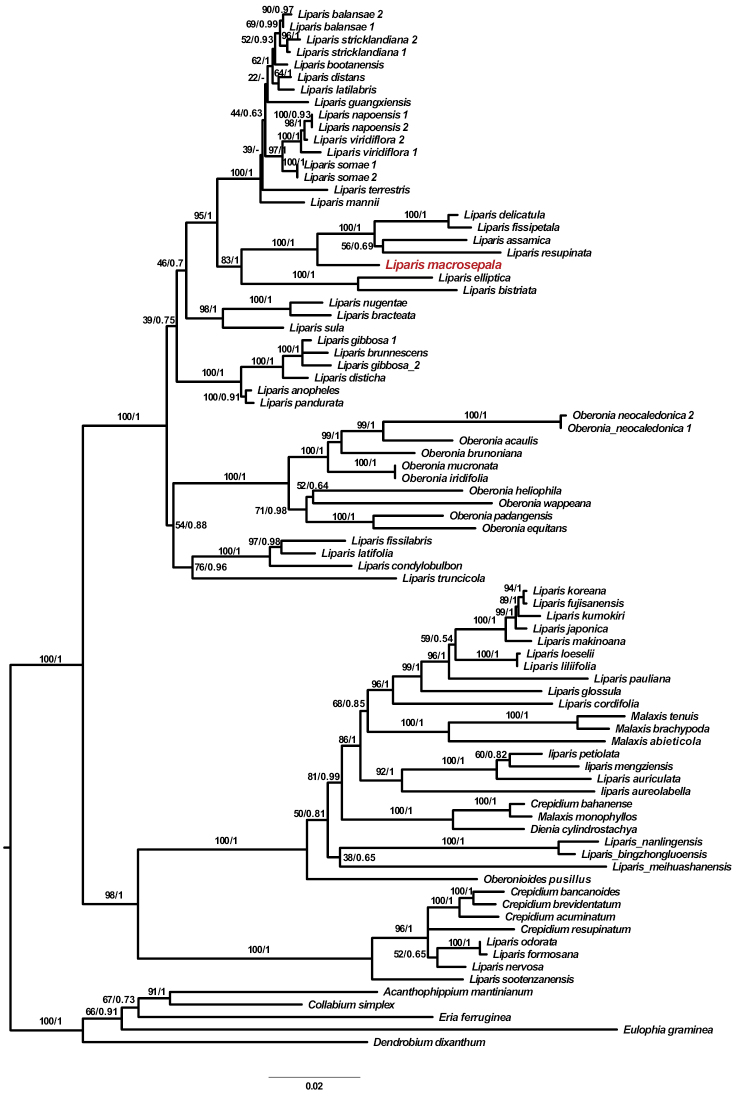
Maximum Likelihood tree of *Liparis* and its allied genera in subtribe Malaxidinae inferred from the combined analysis of nrITS and *matK*. ML bootstrap values (ML_BP_)/Bayesian posterior probabilities (PP) are indicated above the branches, respectively. The sectional taxonomy of *Liparis* follows Garay and Romero-Gonzalez (1999) and Li et al. (2020).

The length of nrITS matrix was 792 bp including 262 parsimony-informative sites and for *matK*, the length and parsimony-informative sites were 1443 bp and 120, respectively. Both analyses (MP and BI) recovered similar relationships. The ML tree with bootstrap percentages, on which the posterior probabilities from the BI analysis were also indicated, is shown in Fig. 1.

The phylogenetic analyses indicate that *Liparis* is not monophyletic, being mingled with species of other genera of Malaxideae. This result agrees with what was found in previous studies (Cameron 2005; Margońska et al. 2012; Tang et al. 2015; Li et al. 2020; Kumar et al. 2022). The new species, henceforth referred to as *Liparismacrosepala* Z.W. Wang, Y. Zhang & W.C. Huang, is grouped with species in *Liparis sect. Cestichis* Thouars ex Pfitzer as the sister of a clade consisting of *L.delicatula* Hook.f., *L.fissipetala* Finet, *L.assamica* King & Pantl. and *L.resupinata* Ridl.

### ﻿Morphological comparisons

*Liparis* is defined as species with racemose inflorescences, resupinate lip lacking a spur, column without a conspicuous foot and four pollinia in two pairs with small viscidium, but no caudicle. The morphology of *Liparismacrosepala* is in accordance with the characteristics of sect. Cestichis like the slightly flattened, narrowly winged rachis with alternating bracts. The morphological characters can distinguish *Liparismacrosepala* from its close relatives *L.delicatula*, *L.fissipetala*, *L.assamica* and *L.resupinata*.

### ﻿Taxonomic treatment

#### ﻿ Liparis macrosepala

Taxon classificationPlantaeAsparagalesOrchidaceae

Z.W. Wang, Y. Zhang & W.C. Huang sp. nov.

81BDBCAD-1560-54AE-829A-15F82007C187

urn:lsid:ipni.org:names:77306143-1

**Figure 2.**
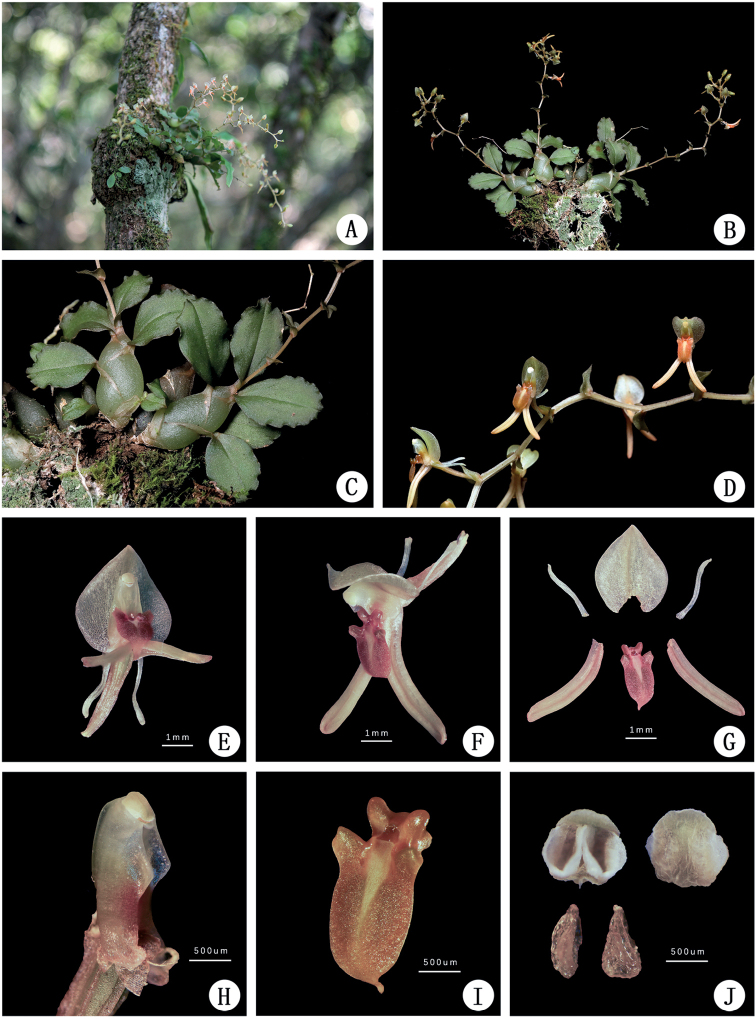
Morphology of *Liparismacrosepala*. **A** plants in situ **B** flowering plant **C** pseudobulbs and leaves **D** inflorescence **E** flowers, front view **F** flowers, side view **G** perianth dissection **H** column from side **I** lip in oblique view **J** anther cap and pollinia. Photographs by Weichang Huang.

**Figure 3.**
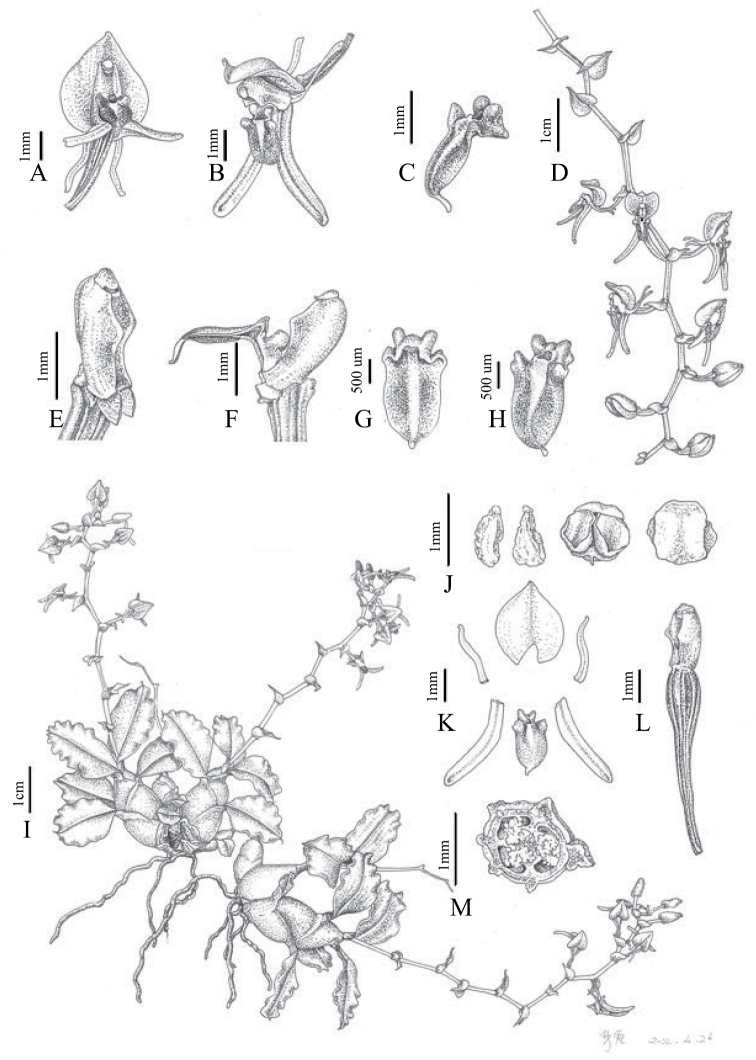
*Liparismacrosepala***A** flower, front view **B** flower, side view **C** lip, side view **D** inflorescence **E** column, side view **F** lip and column, side view **G** lip, back view **H** lip, front view **I** flowering plant **J** pollinia and anther cap **K** perianth dissection **L** column and ovary, oblique view **M** ovary, transection. Drawn by Lan Yan.

Figs 2 , 3 Chinese name: 大萼羊耳蒜

##### Type.

China. Yunnan Province (云南), Xishuangbanna (西双版纳), Mengla County (勐腊县) epiphyte on the tree trunk, 1620 m elev., 23Nov 2021, Zhengwei Wang, Xiaochen Li, Yu Zhang& Zhijin Wu, WZW04247 (holotype: CSH!)

##### Diagnosis.

*Liparismacrosepala* is characterised by the ovoid-fusiform, slightly compressed pseudobulbs with 4 or 5 alternate leaves on their apical half, these with slightly crispate margins, dorsal sepal ovate with cordate base, broadly elliptic, ca. 4 mm long, 2 callus-shaped and thickened folds, base with 2 oblong lobes on both sides, centrally with 1 thickened, concave callus, column with a single pair of arcuate wings.

Epiphytic herbs. Roots slender, flexuose. Pseudobulbs clustered, ovoid-fusiform, slightly compressed laterally, 1–2 × 0.5–1 cm, upper half with 4–5 widely spaced leaves. Leaf blade ovate-oblong, 1.8–2.3 × 0.8–1.2 cm, apex acuminate, base contracted into a short petiole, articulate, margins of their apical half slightly crispate. Peduncle 7–10 cm long, with several sterile bracts 2–5 mm long; raceme with 7–10 flowers arranged in zigzag manner. Floral bracts broadly ovate with cordate base, 2–3 × 1–1.5 mm, acute. Flowers greenish-orange; pedicel and ovary ca. 7 mm long. Dorsal sepal broadly ovate with cordate base, 3.2–5 × 3–3.6 mm, 1-veined, abaxially carinate, apex acute; lateral sepal oblong-ovate or ovate-lanceolate, 5–6 × ca. 0.6 mm long, abaxially slightly carinate. Petals narrowly linear, 3–4 × ca. 0.2 mm; lip elliptic, 2–3 × ca. 1 mm, apex apiculate, base bearing a bituberculate callus, then expanded on each side into a thickened, folded, rounded lobe, with 1 excavation with raised margins between the lobes. Column straight, ca. 2 mm long, with a pair of subtriangular, obtuse wings on each side near the middle and a ridge on the back of the column. Anther cap hemispherical, pale yellow; pollinia 4 in 2 pairs with one pollinium of each pair smaller than the other, waxy, brownish, with minute apical viscidium.

Phenology: Flowering in November–December.

##### Distribution and habitat.

It is found on tree trunks on a limestone ridge-top evergreen broad-leaved forest at an elevation of 1500–1700 m in Mengna County, Xishuangbanna Autonomous Prefecture, Yunnan Province, People’s Republic of China. The habitat presents a tropical monsoon climate.

##### Etymology.

The species epithet refers to the large and conspicuous dorsal sepal of the flower.

##### Taxonomic notes.

*Liparismacrosepala* differs from *L.delicatula* in its 4 to 5 leaves with slightly crispate margins on their apical half and single pair of wings on the column. Its entire, not Y-shaped petals and sessile lip (i.e. without a claw) easily distingush *L.macrosepala* from *L.fissipetala*. The dosal sepal of *L.assamica* is narrowly ovate-oblong, in contrast with the heart-shaped dorsal sepal of *Liparismacrosepala. Liparisresupinata* is distinguished from *L.macrosepala* by its 10–50-flowered raceme and the column with a single pair of broad wings, each with a retrorse thread. The main differences between these closely-related species, according to our phylogenetic analyses, are summarised in Table 2.

**Table 2.** Comparison of *L.macrosepala* and related species.

Characters	*L.delicatula*	*L.fissipetala*	*L.assamica*	*L.resupinata*	*L.macrosepala*
Pseudobulbs	oblong or cylindrical-fusiform 5–9 3–5 mm	ovoid, 8–10 mm long	ovoid-fusiform, slightly compressed 1.5–2.5 cm × 6–10 mm	subcylindrical or ± spindle-shaped, 1.8–5 cm × 3–6 mm	ovoid-fusiform, slightly compressed, 1–2 cm × 0.5–1 cm
Leaf	2 or 3, margin flat	3 or 4, strongly crisped-margined	3 or 4, apical half slightly crisped-margined	3 or 4, margin slightly serrate	4 or 5, apical half slightly crisped-margined
Scape	2–5 cm, several to 10-flowered, flowers white	5–10 cm long, with 10–15 flowers, flowers yellow,	10–13 cm, more than 10-flowered, flowers orange	7–18 cm, 10–50-flowered, flowers pale green or greenish-yellow	7–10 cm, more than 10-flowered, flowers greenish-orange
Bracts	ovate-lanceolate, 2–3 mm	ovate-lanceolate, 1.5–3.5 mm	lanceolate, 2–3 mm	lanceolate, 3–5 mm	broadly ovate, 2–3 mm
Dorsal sepal	ovate-oblong, 2.5–3 × 1.5–1.8 mm	oblong-lanceolate, 3–4 × 0.8–1 mm	narrowly ovate-oblong, 4.8–5.8 × ca. 1.6 mm	oblong or elliptic-oblong, ca. 4 × 1.8 mm	broadly ovate, ca. 3.2–5 ×3–3.6 mm
Petals	narrowly linear-lanceolate, 2.5–3 × ca. 0.5 mm, entire	narrow linear, 4–5 mm long, Y-shaped	narrowly linear, 5–5.5 × ca. 0.7 mm, entire	narrowly linear, ca. 3.5 × 0.3 mm, entire	narrowly linear, 3–4 × ca. 0.2 mm, entire
Lip	broadly elliptic or orbicular, ca. 2.5 mm, base with an orbicular, auriculate, callus-shaped fold on either side, with a concave callus near base	epichile broadly oblong or subsquare, 1.5–2 × 1–1.5 mm, base with two auricles on both sides; claw short, with a fleshy callus centrally near base	broadly obovate-oblong, ca. 4 × 2.7 mm, with two callus-shaped thickened folds, two suborbicular lobes on both sides, centrally with one concave callus near base	broadly elliptic-oblong or broadly ovate-oblong, 2.5–3 mm, with two lateral splits below middle; two suborbicular lobes, centrally with one bilobed callus near base	broadly elliptic, ca. 2–3 mm long, two callus-shaped and thickened folds, base with two oblong lobes on both sides, centrally with one bituberculate callus near base
Column	ca. 2.2 mm, two pairs of wings	ca. 1.5 mm, broadly winged with two horn-like appendages	ca. 2 mm, two pairs of wings	ca. 2.8 mm, a pair of wings, each with a retrorse thread	ca. 2 mm, a single pair of subtriangular wings

##### Conservation assessment.

The new species was found in a ridge-top evergreen broad-leaved forest on a limestone mountain. Despite numerous surveys in the areas, only six mature individuals were found without fruits or evidence of cross-pollination.

This extremely small effective population occurs in a touristic zone which is a serious threat to the survival of the species. Consequently, the species can be assessed as Critically Endangered (CR, D), based on current information and following IUCN guidelines (IUCN 2019).

## Supplementary Material

XML Treatment for Liparis macrosepala
